# Uterine cervical stenosis: from classification to advances in management. Overcoming the obstacles to access the uterine cavity

**DOI:** 10.1007/s00404-023-07126-1

**Published:** 2023-07-10

**Authors:** Salvatore Giovanni Vitale, Maria Chiara De Angelis, Luigi Della Corte, Stefania Saponara, Jose Carugno, Antonio Simone Laganà, Péter Török, Raffaele Tinelli, Tirso Pérez-Medina, Sinem Ertas, Bulent Urman, Stefano Angioni

**Affiliations:** 1 Division of Gynecology and Obstetrics, Department of Surgical Sciences, University of Cagliari, Cagliari, Italy; 2 Department of Public Health, School of Medicine, University of Naples Federico II, Naples, Italy; 3 Department of Neuroscience, Reproductive Sciences and Dentistry, School of Medicine, University of Naples Federico II, Naples, Italy; 4 Division of Minimally Invasive Gynecology, Department of Obstetrics, Gynecology and Reproductive Sciences, Miller School of Medicine, University of Miami, Miami, FL USA; 5 Unit of Gynecologic Oncology, ARNAS “Civico–Di Cristina–Benfratelli”, Department of Health Promotion, Mother and Child Care, Internal Medicine and Medical Specialties (PROMISE), University of Palermo, Palermo, Italy; 6 Department of Obstetrics and Gynecology, Faculty of Medicine, University of Debrecen, Debrecen, Hungary; 7 Department of Obstetrics and Gynecology, “Valle d’Itria” Hospital, Martina Franca, Taranto, Italy; 8 Department of Obstetrics and Gynecology, University Hospital Puerta de Hierro Majadahonda, Autónoma University of Madrid, Madrid, Spain; 9 Department of Obstetrics and Gynecology, Koc University School of Medicine, Istanbul, Turkey

**Keywords:** Cervical stenosis, Hysteroscopy, Infertility, Therapy

## Abstract

**Background:**

To date hysteroscopy is the gold standard technique for the evaluation and management of intrauterine pathologies. The cervical canal represents the access route to the uterine cavity. The presence of cervical stenosis often makes entry into the uterine cavity difficult and occasionally impossible. Cervical stenosis has a multifactorial etiology. It is the result of adhesion processes that can lead to the narrowing or total obliteration of the cervical canal.

**Purpose:**

In this review, we summarize the scientific evidence about cervical stenosis, aiming to identify the best strategy to overcome this challenging condition.

**Methods:**

The literature review followed the scale for the quality assessment of narrative review articles (SANRA). All articles describing the hysteroscopic management of cervical stenosis were considered eligible. Only original papers that reported data on the topic were included.

**Results:**

Various strategies have been proposed to address cervical stenosis, including surgical and non-surgical methods. Medical treatments such as the preprocedural use of cervical-ripening agents or osmotic dilators have been explored. Surgical options include the use of cervical dilators and hysteroscopic treatments.

**Conclusions:**

Cervical stenosis can present challenges in achieving successful intrauterine procedures. Operative hysteroscopy has been shown to have the highest success rate, particularly in cases of severe cervical stenosis, and is currently considered the gold standard for managing this condition. Despite the availability of miniaturized instruments that have made the management of cervical stenosis more feasible, it remains a complex task, even for experienced hysteroscopists.

## Introduction

The cervical canal is the passageway to the uterine cavity. If stenotic, it will impair the access, thus leading to failure to perform the intended hysteroscopic procedure or to complications such as uterine perforation, cervical laceration, or the creation of a false passage [1, 2]. In a recent series, Bettocchi et al. showed that the main reasons for incomplete or failed hysteroscopies were pain and cervical stenosis [3]. Recent technological innovations, along with increased operator experience and optimal pain management, have made it possible to overcome cervical stenosis with the use of office hysteroscopy, significantly reducing the rate of failed procedures and the need for general anesthesia [4]. With the growing role of office hysteroscopy in the diagnosis and management of uterine pathology and acknowledging the impact of cervical stenosis on the success of the procedure, we summarized the available evidence about stenosis of the uterine cervix, starting from its classification up to innovative therapeutic strategies implemented to overcome the present challenges.

## Materials and methods

We adhered to the quality standards for narrative reviews, as defined and quantified by the scale for the quality assessment of narrative review articles (SANRA) [5]. The relevant publications were identified after a systematic query of PubMed, Google Scholar, Scopus, Web of Science, and research registers (such as Clinicaltrials.gov) complemented by cross-checking the reference lists. We used a combination of the search terms “cervical stenosis”, “vaginoscopy”, “hysteroscopy”, “pathogenesis”. No language restriction was applied. All articles, published between 1983 and March 2023, describing the management of cervical stenosis were considered eligible for review. Relevant aspects of each article were recorded and commented on, with particular attention to the type of treatment applied and described outcomes.

## Uterine cervical stenosis

### Definition and classification

**Fig. 1:**
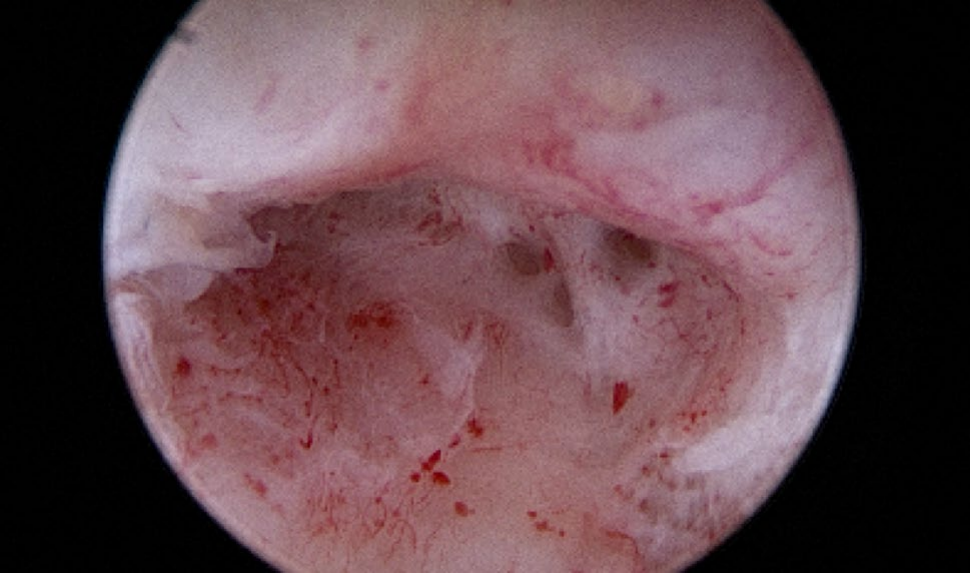
Uterine cervix with moderate fibrotic synechia during hysteroscopic evaluation

Although there is no consensus on the definition of cervical stenosis, it could be defined as a cervix with an obliterated cervical ostium and/or cervical canal that requires particular maneuvers for the introduction of the hysteroscope in order to access the uterine cavity (Fig. 1). According to Baldauf’s definition, cervical canal stenosis occurs when the cervical canal does not allow the passage of a 2.5 mm Hegar cervical dilator [6], while external cervical os (ECO) stenosis has been defined as when the diameter of the ECO is less than 4.5 mm [7].

The true incidence of cervical stenosis cannot be estimated because most patients are asymptomatic and this condition is only diagnosed in those with the indication of evaluation of the uterine cavity. In a series of 31,052 office hysteroscopies, Bettocchi et al. identified cervical stenosis in 32.7% of the patients: among them, 70.1% were postmenopausal, and 29.9% were of reproductive age. Moreover, the frequency of the different types of stenosis differed according to the age groups [3]. ECO stenosis was found more frequently in premenopausal than in postmenopausal women, while internal cervical os (ICO) stenosis were more frequent in menopausal women, often representing a challenge for even the most experienced endoscopist [3].

**Fig. 2:**
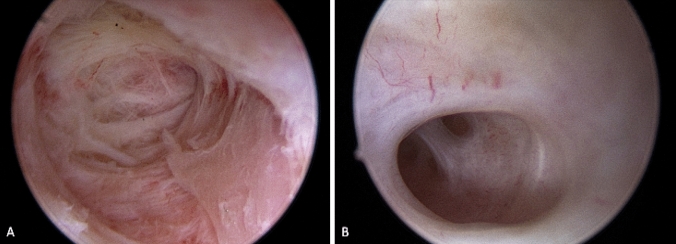
**A** Severe fibrotic synechiae completely distorting the cervical canal; **B** Mild synechia on the left lateral wall

Even though synechiae are frequently encountered at the level of the internal cervical ostium, they may also be distributed all along the cervical canal (Fig. 2A) together with obliteration of the ICO [8] (Fig. 2B).

Table 1 shows the classification proposed by Bettocchi in 2016 that recognizes four types of cervical stenosis depending on the structure or structures affected (ECO, cervical canal, ICO) [3].

**Table 1:** Classification proposed by Bettocchi in 2016 that recognizes four types of cervical stenosis depending on the structure or structures affected (ECO, cervical canal, ICO) [3]

Type	Structure affected
I	ECO stenosis
II	Cervical canal and ICO stenosis
III	ICO stenosis
IV	ECO and ICO stenosis

*ECO* external cervical os, *ICO* internal cervical os

### Pathogenesis and clinical implications

Cervical stenosis occurs as a result of adhesions involving the internal wall of the cervical canal, causing its narrowing, distortion or complete obliteration [8]. It may be congenital or acquired, and due to procedures performed on the cervix [9], endometrial ablation performed without sparing the isthmic uterine segment [10], diathermal loop excision of cervical pathologies [6], conization of the cervix using “cold knife” techniques or cervical trauma [11, 12], infection, cervical or endometrial cancer, radiation, vaginal infections, or mass effect, due to the presence of Nabothian cysts (Figs. 4, 5) or large leiomyomas of the cervix [13]. In addition, atrophic and/or dystrophic processes resulting from estrogen deficiency after menopause can induce the progressive narrowing of the cervical canal [14, 15].

Congenital cervical stenosis is much rarer than acquired. Accumulating evidence suggests a correlation between the presence of stenosis of the ECO and endometriosis in women with chronic pelvic pain [7].

Nulliparity, previous endometrial curettage, tamoxifen use [16], and treatments for cervical dysplasia, such as cervical conization [6, 11, 12], cryotherapy, and colposcopic biopsies are common risk factors for cervical stenosis [13].

Patients are frequently asymptomatic, especially when the cervical stenosis is incomplete, allowing drainage of menstrual blood [13, 17]. Symptoms may include pelvic pain and severe dysmenorrhea [13, 17]. When cervical stenosis is complete, it may cause hematometra, hydrometra or pyometra, presenting with severe pain [14, 15]. In women of reproductive age, cervical stenosis may cause infertility and secondary amenorrhea, and in some cases, it can lead to retrograde menstruation [7].

### Implications

Cervical stenosis may be a reason for infertility. In 2011, Plante et al. reported cervical factors as a cause of infertility in 40% of cases [18]. In 2020, Izhar et al. reported that cervical factor was implicated in around 5% of couples presenting with infertility [19]. They stated that cervical factor could be caused by cervical stenosis, which frequently remains undiagnosed in couples undergoing the infertility workup, according to criteria suggested by European Society of Human Reproduction and Embryology (ESHRE) [20] and American College of Obstetricians and Gynecologists (ACOG) [21]. This happens because the fertility algorithm does not involve routine evaluation of the uterine cavity, therefore, the presence of cervical stenosis is diagnosed only when intrauterine insemination or embryo transfer is attempted. There are several ways in which a stenotic cervix can lead to infertility: it can prevent intrauterine deposition of semen and can interfere with the production of cervical mucus, which can make it difficult for sperm to move and survive [19, 22, 23]. Moreover, cervical stenosis might cause menstrual problems such as reduced menstrual flow, prolonged or irregular periods, dysmenorrhea and amenorrhea [13, 17]. Blocked or held-back menstrual bleeding can lead to uterine inflammation and an increased risk of endometriosis [7]. Finally, it should be noted that the embryo transfer can be impaired by the presence of cervical stenosis [24]. For this reason, some clinicians suggest including hysteroscopy in the diagnostic work-up of the infertile woman [10, 25–27]. Additionally, embryo transfers performed under ultrasound guidance would improve conception rates by ensuring correct tip position inside the uterine cavity and proper embryo deposition in the presence of cervical stenosis [28].

### Diagnosis

Hysteroscopy is the gold standard for the diagnosis and treatment of cervical stenosis [29, 30]. The procedure allows the visualization of the cervical canal under magnification, enabling evaluation of the extent, localization and consistency of the adhesions [30].

There are no imaging techniques suited to evaluate the patency of the cervical canal or to identify adhesions. Cervical stenosis is frequently diagnosed during routine clinical practice, when at the time of performing a pap smear the ECO is closed and not accessible to sampling of the cervical canal with the cytobrush. Transvaginal ultrasound may be useful in the diagnosis of complications as a result of cervical stenosis such as hematometra, pyometra, or hydrometra [14, 15]. The detection of intrauterine fluid collection in postmenopausal women could also be a consequence of cervical stenosis [14, 15].

## Treatment options

The main objective of both medical and surgical treatments for cervical stenosis is to restore the patency of the cervical canal. These treatments are indicated in cases where symptomatic obstructions cause complications such as hematometra or pyometra [14, 15]. Additionally, in menopausal patients with occasional ultrasound findings of intracavitary fluid due to cervical stenosis and an endometrial thickness > 3 mm, treatment of the stenosis is recommended to evacuate the fluid, inspect the endometrial cavity, and perform biopsies due to the risk of endometrial malignancy[14, 15, 31–33].

### Non-surgical treatment

They include several strategies

- *Laminaria* They are composed of dried marine algae that extract liquid from the cervical tissue, expanding its diameter. Therefore, they act as osmotic dilators that reach their maximum effect in 24 h. The insertion of laminaria stems before the procedure is intended to simplify the cervical dilation [34, 35]. However, laminaria stems can increase the risk of infection and placement of the stem requires at least some degree of dilatation of the external cervical os [34–36].
- *Misoprostol* It is a prostaglandin E_1_ analogue, that ease cervical dilation through its estrogen-mediated effects on the cervix. It can be used orally, sublingually, or vaginally. Randomized control studies that have compared misoprostol with placebo in nulliparous premenopausal women undergoing hysteroscopy suggested a potential benefit of misoprostol when a difficult-to-access cervix is suspected. It was observed that doses of 400 µg orally or 200 µg vaginally, taken at least 9–12 h before the procedure, may be beneficial before hysteroscopy, although vaginal administration is associated with more menstrual-like pain and vaginal bleeding than when administered using the oral route [37, 38]. In postmenopausal women and those treated with gonadotropin-releasing hormone analogs, misoprostol has a decreased effect since prostaglandins require estrogen to generate their cervical ripening effects, and postmenopausal patients are in a hypoestrogenic state [39]. Thomas et al. in a randomized study in postmenopausal women, administered 400 µg of misoprostol or placebo orally 12 and 24 h before operative hysteroscopy, reporting that misoprostol requires a longer duration to achieve clinical efficacy [40]. In a randomized trial, Kant et al. administered 200 µg of misoprostol into the vagina 12 h before outpatient hysteroscopy, and they demonstrated a statistically significant difference between the study group and the control group receiving placebo when evaluating pre-procedure cervical canal width (7.7 ± 1.7 vs. 4.5 ± 1.8 mm), the number of women requiring additional dilation (7/25 vs 22/25) and the time required for dilation (4.7 ± 8 vs 20.6 ± 9.3 s) [41]. More recently, Nandhini et al. have added further evidence to the benefits of misoprostol as a cervical priming agent in women undergoing diagnostic hysteroscopy for abnormal uterine bleeding. In this randomized trial involving 122 women, the study group received a 200 μg vaginal dose of misoprostol 3 h before vaginoscopic hysteroscopy, while the control group received no drug. The results showed that a higher percentage of women in the misoprostol group experienced easy or very easy cervical entry (75.41% vs. 27.87% in the control group). Furthermore, the median pain score and the median procedural entry time were significantly lower in the misoprostol group than the control group [42]. In an observational comparative study, Casadei et al. highlighted the relevance of estrogen pretreatment in allowing the effect of misoprostol on cervical ripening [43]. Intravaginal misoprostol and laminaria stems have very similar benefits before hysteroscopy in women with a stenotic cervix; nevertheless misoprostol is easier to use [44].
- *Mifepristone* In the literature, there are conflicting studies on the effects of mifepristone on the cervical preparation; therefore, there are no clear recommendations regarding its use. Indeed, Gupta and Johnson demonstrated benefits when mifepristone 600 mg was administered 48 h before dilation and curettage in postmenopausal women [45]; however, Ben-Chetrit et al. did not show any advantage using mifepristone 200 mg administered 30 h before office hysteroscopy [46].
- *Dinoprostone* Conflicting results have been reported on the effectiveness of Dinoprostone vaginal prostaglandin E2. In one trial, vaginal dinoprostone was shown to be more effective in ripening the cervix than vaginal misoprostol or placebo in women undergoing office hysteroscopy [47]; however, Preutthipan and Herabutya, in a randomized prospective study, found that vaginal misoprostol was more effective in cervical ripening compared with vaginal dinoprostone in nulliparous women before operative hysteroscopy [48].

### Surgical treatment

**Fig. 3:**
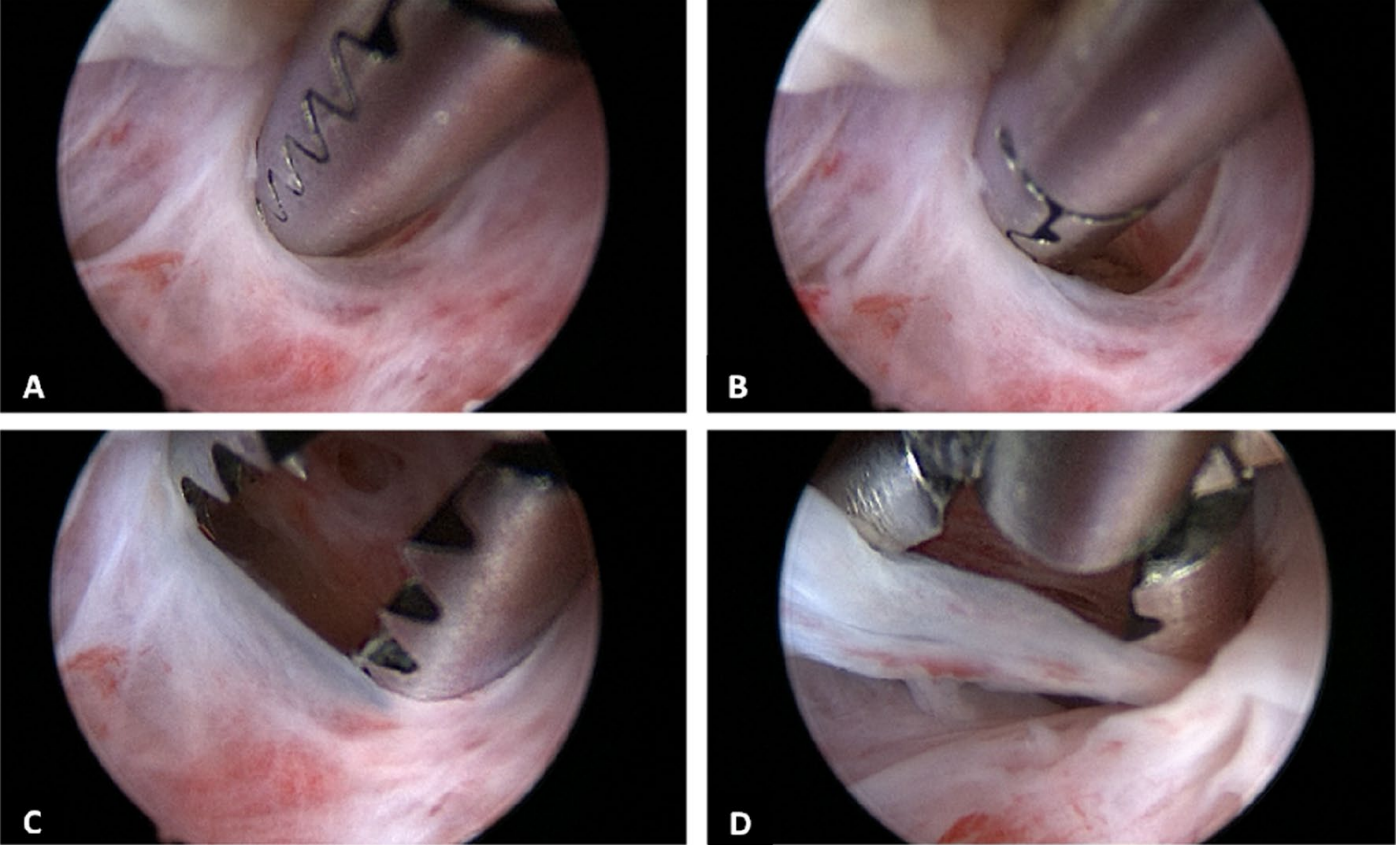
Technique of adhesiolysis using a 5 Fr grasper. Since moderate fibrotic adhesions hide a clear passage through the cervix, the 5 Fr grasper is passed into the fibrotic tissue **A**, creating a small opening of the way **B**. With a continuous opening and closing of the grasp jaws, the lumen is further enlarged allowing the hysteroscope to pass into the uterine cavity **C–D**

**Fig. 4:**
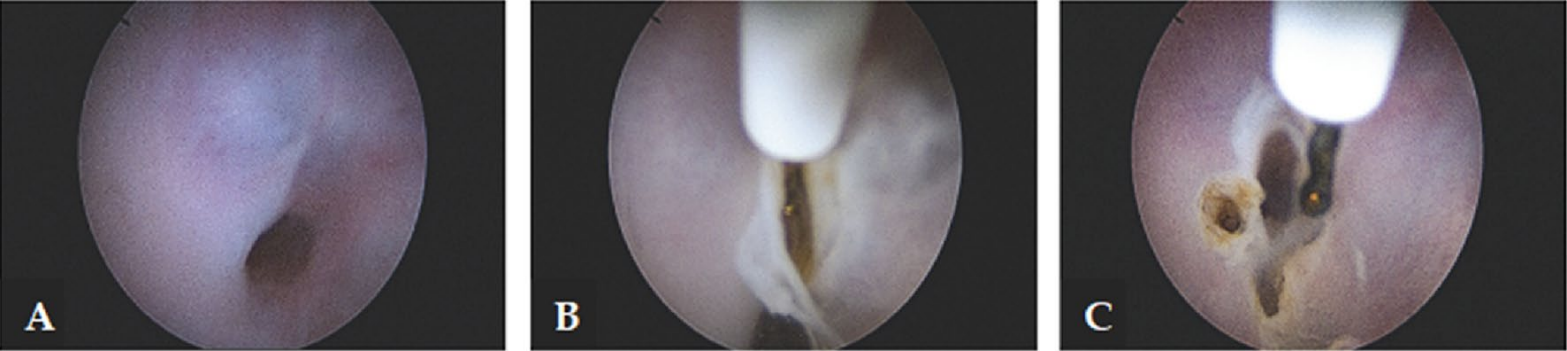
Severe stenosis of cervical canal with a keyhole-shaped external cervical os **A**. A 5-Fr bipolar electrode is carefully inserted into the stenotic ostium **B**, then a cut is made along the four cardinal points performing a star-shaped incision in order to create an adequate access to the uterine caity **C**

**Fig. 5:**
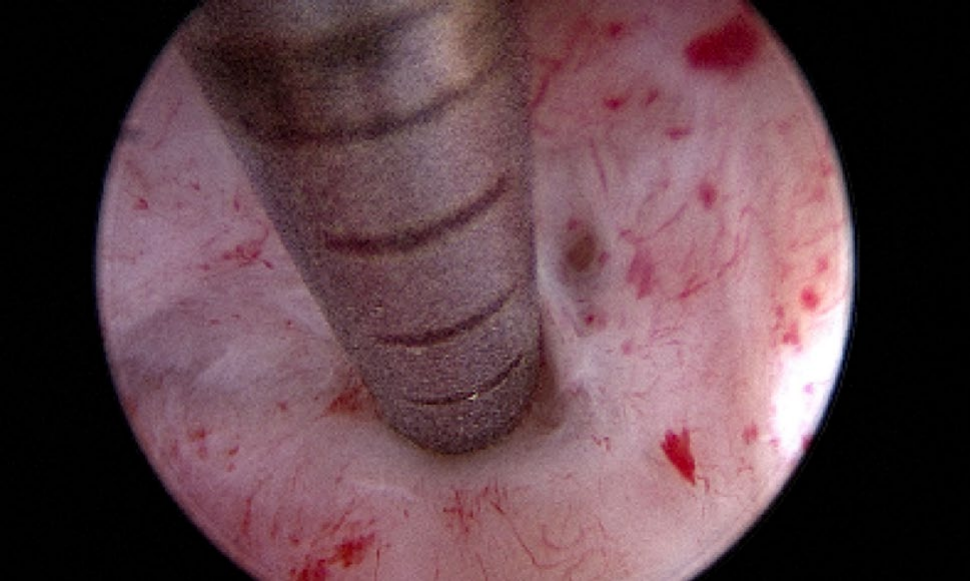
5 Fr graduated intrauterine palpator used to perform the lysis of severe fibrotic cervical adhesions

- *Cervical dilators* A mechanical option to dilate the cervix is with using Hegar or Pratt dilators. [49, 50]. However, this method is associated with the risk of several complications, such as the creation of a false passage or uterine perforation [49, 50]. Vasopressin has been reported to reduce the force required for entry in cases of cervical stenosis when injected into the cervix before the procedure [51]. Due to its potential cardiorespiratory side effects, vasopressin should only be used for this purpose under cardiac monitoring.
- *Hysteroscopy* This minimally invasive procedure represents the gold standard approach for the management of patients with cervical stenosis [29, 30]. With direct visualization, it is possible to navigate the cervix, reducing the risk of injury. Also, the vaginoscopic approach is particularly useful in the setting of a stenotic cervix because it uses hydro-dilation of the cervical canal allowing to introduce the hysteroscope [52]. Moreover, when performing in-office hysteroscopy without anesthesia, the feedback from the patient guides the procedure, as the fibrous scar tissue is painless to go through, but when deviating from the cervical canal, there is increased pain suggesting the possibility of creating a false passage [53]. In 2016, Bettocchi et al. described 4 useful hysteroscopic techniques to overcome cervical stenosis [3]. In detail, when only filmy adhesions are found, blunt lysis of adhesions can be performed using the distal tip rotating the hysteroscope. In cases of moderate adhesions, the use of miniaturized instruments to clear the passage into the cervix could be helpful. Therefore, 5 Fr graspers could be introduced into the stenotic cervical canal and rotated to a degree that the opened jaws are in line with the transverse plane of the cervical canal. Gradually, the endocervical canal becomes visible so that the hysteroscope can be advanced (Fig. 3). Also, blunt 5 Fr scissors can be used to transect dense adhesions [54, 55]. Finally, when in presence of complete obliteration of the cervical canal or a keyhole-shaped ECO, a bipolar electrode can be used to perform a star-shaped incision. The cuts are placed in a radial pattern creating an adequately sized ECO (Fig. 4) [3, 55]. Using the vaginoscopic approach, it is possible to identify the external os in those cases where it is not easily detectable with the naked eye; in particular, by modifying the intravaginal pressures, the protrusion of the cervix into the vagina is determined, making it possible identify the “blue behind the white” sign (translucent endocervical mucus that makes the ECO shine) allowing to identify the exact location of the ECO [55]. Among the miniaturized instruments that can be used to overcome cervical stenosis, the 5 Fr graduated intrauterine palpator should be mentioned [56]. This instrument, originally designed to accurately measure the length of the resected uterine septa after hysteroscopic metroplasty, can be used to apply pressure to the cervix in order to identify the opening of the external cervical os (Fig. 5).; this opening can be subsequently expanded with 5 Fr bipolar electrode or the “grasping” forceps, as is done in cases of mild stenosis [55]. Managing severe cervical stenosis in office setting is also possible using Tissue Removal Systems. Salari et al. in a video article demonstrated the efficacy and safety of this new approach, minimizing the potential risk of uterine perforation and the creation of a false track [57]. Recent advancements have also resulted in the development of small and adaptable tissue removal systems that can be used through a 5fr working channel, further enhancing the efficacy and safety of procedures in an office setting [58]. To date, the standard resectoscopic approach is reserved only for cases where outpatient treatment fails. The narrow cervical canal can be entered using the 26 Fr resectoscope [24, 59].

### Tips and tricks

A normal cervix can become a barrier if hysteroscopy is performed incorrectly. The impact of the tip of the hysteroscope on the wall of the cervical canal causes pain and bleeding, which makes it difficult to visualize the cervical canal and, therefore, entering the uterine cavity.

This is the reason why, the combination of proper technique and endoscopic experience, along with optimal pain management [60, 61] makes it possible to overcome even severe cervical stenosis with the use of office hysteroscopy (Table 2).

**Table 2:** Key-points for management of cervical stenosis

Step	Key-points
1	Identify patients at risk for cervical adhesions
2	Perform adequate patients’ counseling
3	Use the “vocal local” approach (Talk to the patient during the procedure)
4	Choose the adequate instrumentation. Favor miniaturized instruments when available
5	Apply pressure with the hysteroscopic grasper or intrauterine palpator in order to identify the opening of the ECO
6	Increase the pressure of the fluid distension medium
7	Ensure adequate pain management
8	When navigating the stenotic cervix, move back the hysteroscope to obtain a panoramic view of the cervical canal
9	Use of ultrasound guidance when available
10	Consider the use of an ultrasound-guided trocar-containing 18-gauge needle

*ECO* external cervical os

First, adequate counseling before starting the procedure is important. In particular, women with risk factors for cervical stenosis should be informed that, although hysteroscopic procedures are generally quick and the pain is usually well tolerated, some discomfort should be expected when cervical stenosis is encountered. Talking to the patient during the procedure describing what is happening, informing her whether there is cervical stenosis, and explaining the procedure in detail, may reduce the discomfort [53, 60, 62].

Choosing the adequate instrumentation is mandatory in case of cervical stenosis. Smaller diameter hysteroscopes are the best to overcome cervical stenosis, offering an optimal view with minimal discomfort for the patient. In 2016, Salari et al. proposed the use of a small hysteroscopic morcellator that simultaneously shaved and removed the fibrous cervical tissue in order to overcome the cervical stenosis [57]. Despite it is recommended to navigate through the endocervical canal using the lowest pressure of the distension medium, it may become necessary to increase the fluid pressure when attempting to overcome cervical stenosis [63]. Adequate pain management is mandatory in cases of cervical stenosis. It is well known that the cervical canal is the most innervated part of the uterus, as well as the most challenging to approach with the hysteroscope. Softening the cervix with the administration of vaginal misoprostol or intracervical prilocaine, could be helpful in cases of cervical adhesions [64]. However, there is no consensus in the literature on the use of pharmacologic agents for pain management. The Royal College of Obstetricians and Gynaecologists (RCOG) guidelines recommend taking standard doses of non-steroidal anti-inflammatory drugs (NSAID) around 1 h before the procedure [65], while ACOG and Italian Society of Gynecology and Obstetrics (SIGO) guidelines state that there is no clinically significant difference in the effectiveness of analgesia regimens when compared to placebo [29, 66].

Another tip for managing cervical stenosis is the use of transabdominal or transrectal ultrasound as an intraoperative guidance in order to minimize the risks of perforation or other complications [67].

In severe cases, it is recommended to fill the bladder so concomitant abdominal ultrasound can be performed, allowing the surgeon to monitor the path while introducing the hysteroscope or other instruments into the uterine cavity [67]. A recent study reported the use of a 5.7 Fr coaxial catheter with an outer echogenic sheath and an inner 0.018-in diameter guidewire with a coude tip, which is first introduced into the uterine cavity and visualized with ultrasound [68]**.** After placing the catheter inside the uterine cavity, the surgeon advances the hysteroscope following the guide of the catheter. Another method that has been described to overcome difficult hysteroscopic access to the uterine cavity is the use of an ultrasound-guided trocar-containing 18-gauge needle that punctures the central area of the cervix [67]. Further access can be reached by passing a floppy-tip wire through the needle and coiling it into the uterine cavity, confirming intrauterine placement using an endorectal probe. Later progressive dilators are passed over the wire, so a channel to pass instrumentation is created [67].

## Preventive strategies

Cervical stenosis is more likely to occur among menopausal and nulliparous women in which case it is difficult to prevent. Instead, different strategies have been proposed to prevent the recurrence of cervical adhesions after surgery, such as conization of the cervix.

Luesley et al. described in 33 patients a novel approach involving cervical stenting, sutured immediately after surgery and left in place for 2 weeks for the prevention of cervical stenosis [69]. The device is shaped like a hollow funnel, which fits into the cervical canal: its body contains multiple drilled holes to allow drainage of cervical stromal exudate, the tapered end is placed in the residual canal, and the other end contains eight drilled holes to allow the anchoring of sutures. At follow-up 6 months later, the rate of cervical stenosis was 6% [69]. In a randomized clinical trial by Vieira M. de A. et al., the authors evaluated a new device for the prevention of cervical stenosis. The device is composed of a cylindrical portion, which makes contact with the external surface of the cervix, and a 4-mm diameter cylindrical rod with a central lumen, which is inserted into the endocervical canal. Their study did not show statistically significant differences, ranging from 4 to 7.3% and 5.8 to 4.3% in the groups with and without the anti-stenosis device to prevent cervical stenosis, respectively [70].

The effectiveness of the Levonorgestrel-releasing Intrauterine Device (LNG-IUD) for the prevention of cervical stenosis has been evaluated since the dilation required for the insertion of the device allows menstrual blood to drain. The mechanism of action is also thought to be based on the progesterone effect, which leads to thinning and atrophy of the endometrium, and on suppression of estrogen receptors, which contribute to reducing menstrual bleeding. So, LNG-IUS can be effective in women with dysmenorrhea and cervical stenosis because of its hormonal and mechanical effects. Motegi et al. described the use of the LNG-IUS after cervical dilation surgery for two patients with severe cervical stenosis after uterine cervical conization [71]. Their symptoms improved, and the LNG-IUS was left in place for 5 months after treatment. Further studies with larger numbers of patients are needed to confirm the efficiency of this strategy.

## Conclusions

Access to the uterine cavity through the cervical canal is critical for diagnosing and treating patients with intrauterine pathology. However, cervical stenosis can compromise the accomplishment of the hysteroscopic procedure. Although miniaturized instruments have simplified the management of cervical stenosis, it remains a significant challenge. Therefore, it is essential to avoid curettage because it is ineffective and can make the upcoming hysteroscopic procedure more difficult. Hysteroscopy is the gold standard technique for treating cervical stenosis and should be adopted whenever possible.

## Data Availability

Not applicable.
